# Evaluation of Phytosterols as an Alternative to Cholesterol in Practical Diets on Growth and Nonspecific Immunity of *Litopenaeus vannamei*

**DOI:** 10.1155/2023/7825559

**Published:** 2023-04-17

**Authors:** Yongkang Chen, Zhongchao Pan, Xiaoyue Li, Xinzhou Yao, Guilun He, Shiwei Xie

**Affiliations:** ^1^Laboratory of Aquatic Animal Nutrition and Feed, College of Fisheries, Guangdong Ocean University, Zhanjiang, Guangdong 524088, China; ^2^State key Laboratory of Biocontrol, Guangdong Provincial Key Laboratory for Aquatic Economic Animals and Southern Marine Science and Engineering Guangdong Laboratory (Zhuhai), School of Life Sciences, Sun Yat-Sen University, Guangzhou 510275, Guangdong Province, China; ^3^Guangdong Wei Lai Biotechnology Co., Ltd, Guangzhou 510000, China; ^4^Aquatic Animals Precision Nutrition and High-Efficiency Feed Engineering Research Centre of Guangdong Province, Zhanjiang, Guangdong 524088, China; ^5^Key Laboratory of Aquatic, Livestock and Poultry Feed Science and Technology in South China, Ministry of Agriculture, Zhanjiang 524088, China

## Abstract

This study is aimed at evaluating the effectiveness of phytosterols as an alternative to cholesterol in practical diets of Pacific white shrimp *Litopenaeus vannamei* from the perspective of growth and nonspecific immunity. Five diets were formulated to contain different sterol sources and levels. Two diets were supplemented with 1 g/kg cholesterol (LC (low cholesterol)) or phytosterol (LP (low phytosterol)). Other three experimental diets were supplemented with 2 g/kg cholesterol (HC (high cholesterol)), 2 g/kg phytosterol (HP (high phytosterol)), or mixed sterol source (CP, 1 g/kg cholesterol + 1 g/kg phytosterol), respectively. A total of 750 healthy and uniform-sized shrimp (0.52 ± 0.008 g) were randomly distributed into 5 groups with 3 replicates and fed with the five experimental diets for 60 days. Results showed that the growth performance of shrimp was influenced by the sterol levels and supplementation with 2 g/kg sterol level facilitated the growth of shrimp. The inclusion of phytosterol has a cholesterol-lowering effect on shrimp, as evidenced by a reduction in hemolymph cholesterol and triglyceride contents in the HP group. Besides, supplementation with 2 g/kg phytosterol or mixed sterol sources had positive effects on the hemolymph superoxide dismutase, phenol oxidase, and lysozyme as well as hepatopancreas alkaline phosphatase activities, demonstrating that the nonspecific immunity and antioxidative capacity were improved. In conclusion, phytosterols could be an appropriate alternative to partially replace dietary cholesterol in shrimp feeds. This study preliminarily revealed the effects of different sterol sources and levels on the growth and nonspecific immunity of shrimp and provided a basis for further exploration of the mechanism of phytosterol.

## 1. Introduction

Cholesterol, as one of the essential lipid sources for animals, together with phospholipids and fatty acids, forms the integral component of cell membranes [1]. Also, cholesterol is a nonpolar lipid constituent that acts as a precursor for some crucial growth and reproduction hormones [2]. More importantly, cholesterol is a component of lipoproteins that are involved in the transport and absorption of lipids in animals [3]. Usually, teleost is born with the ability to synthesize cholesterol from acetate via squalene *de novo*. However, although crustaceans are considered a rich source of cholesterol, it is generally accepted that shrimp or other crustaceans cannot synthesize cholesterol *de novo* due to the lack of squalene monooxygenase and lanosterol synthase [4]. Therefore, crustaceans must obtain cholesterol or sterols from their diet to meet the growth, molting, and reproduction requirements.

As a potential alternative for cholesterol, phytosterols are plant-based sterols and mainly constitute *β*-sitosterol, campesterol, and stigmasterol [5]. It has been reported that phytosterols can be a partial or complete substitute for cholesterol in *Litopenaeus vannamei* feed [6]. In a previous study, supplementation of 1.2 g/kg phytosterols in semipurified diets significantly increased the final weight and weight gain rate of *Litopenaeus vannamei* [7]. It is also reported that 1.0 g/kg of dietary phytosterols meets the requirement of *Litopenaeus vannamei* [8]. Generally, phytosterols are thought to reduce plasma cholesterol by inhibiting cholesterol uptake by intestinal cells and promoting cholesterol excretion, which is considered the cholesterol-lowering effect [9]. On the other hand, phytosterols also function as antioxidants and play a positive role in immunity [10, 11]. According to a previous study, dietary phytosterols can alleviate oxidative stress induced by a high-lipid diet in large yellow croaker (*Larimichthys crocea*) by activating the activities of catalase and superoxide dismutase [12]. Further, dietary phytosterols increased plasma total immunoglobulin contents and upregulated the gene expressions of *β-defensin*, *hepcidin antimicrobial peptide 1*, *interleukin-10*, and *interleukin-18* of gilthead seabream (*Sparus aurata*) after the 48 d of feeding trial [13], which implied the immunopotentiation effect of phytosterol.

The objective of this study was to preliminarily evaluate the effect on growth and nonspecific immunity of shrimp after different sterol sources and levels were supplemented in diets. The Pacific white shrimp, *Litopenaeus vannamei*, gaining popularity due to its fast growth rate, high survival, unique taste, and abundance of nutrients, has been employed in this study. With the increasing constituent of plant-based ingredients in practical diets such as soybean meal and soybean oil, farmed shrimp may grow slowly due to a lack of cholesterol [5]. In addition, studies on the immune response of dietary phytosterols have mostly been conducted in the teleost, while the studies on the nonspecific immunity of *Litopenaeus vannamei* are still limited. Since shrimp rely mainly on innate immunity rather than adaptive immunity, dietary phytosterols may have different effects on the immune response of shrimp. Therefore, a feeding trial was conducted to demonstrate the effectiveness of phytosterols in practical diets of shrimp by the comparison of different sterols sources and levels. This study sought to preliminarily explore the effect of phytosterol on the growth and nonspecific immunity of shrimp.

## 2. Material and Methods

### 2.1. Diet Preparation

The cholesterol was purchased from Sigma-Aldrich (USA) and the phytosterol (constitute of 28.1% of campesterol, 17.2% of stigmasterol, 43.9% of *β*-sitosterol) was provided by Guangdong Wei Lai Biotechnology Co., Ltd. (Guangzhou, China). The main protein sources were composed of meat and bone meal, fishmeal, and soy protein concentrate. The lipid sources were made with fish oil, soybean oil, and soybean lecithin. Based on a previous study, the control diet was formulated to contain 1 g/kg cholesterol (LC (low cholesterol)) [7] and phytosterol was used to replace cholesterol in the LP (low phytosterol) diet. In the other three experimental diets, cholesterol supplementation was increased to 2 g/kg (HC (high cholesterol)) and then, cholesterol was partially (CP, 1 g/kg cholesterol + 1 g/kg phytosterol) or fully (HP (high phytosterol)) replaced with phytosterols. To prepare the five diets, all ingredients were ground and sieved through an 80-mesh screen, weighed, and mixed to homogeneity (M-256, South China University of Technology, China) [14]. The 1.5 mm diameter pellets were extruded using a pelletizer (Institute of Chemical Engineering, South China University of Technology, Guangdong, China), heated at 60°C for 30 min, and stored at -20°C before use. The formulation and nutrient levels of the diets are shown in Table 1.

### 2.2. Shrimp Rearing and Experimental Conditions

Juvenile shrimp were obtained from Doumen (Zhuhai, China). The shrimp were fed commercial feed and acclimatized to the experimental conditions before being used. During shrimp distribution, the 750 uniform-sized and healthy shrimp (initial weight 0.52 ± 0.008 g) were divided into 5 groups in triplicate, with 50 shrimp in a 500 L fiberglass tank. Normally, shrimp were fed three times a day at 8 : 30, 13 : 00, and 18 : 00, with a total feeding rate of 3%-8% of shrimp weight per day [15]. In case of extreme weather such as typhoons and thunderstorms, feeding may be reduced or even suspended. The feeding trial lasted for 60 days and was conducted in an outdoor recirculating water culture system. During the experiment, the water quality was continuously detected. The water temperature was kept at 25-32°C, dissolved oxygen >5 mg/L, salinity at 5‰, and continuous ventilation. The pH fluctuated from 7.7 to 8.5. The concentration of ammonia nitrogen was below 0.2 mg/L.

### 2.3. Sample Collection and Analysis

At the end of the feeding trial, shrimp were starved for 24 h to obtain basal metabolic profiles. The shrimp in each tank were counted and weighted to calculate the growth indicators. Eight shrimp from each tank were collected for whole-body composition analysis. The hemolymph was collected from 15 shrimp per tank using a 1 mL sterile syringe and then placed at 4°C for 1 h [16]. After centrifugation at 4°C, 8000 rpm for 15 min, the supernatant was obtained and stored at -80°C before hemolymph biochemical parameters determination. The hepatopancreas was removed from 4 shrimp per tank as a sample pool and stored at -80°C before enzyme activity assay.

The moisture of diets and shrimp was determined by oven drying at 105°C according to the standard of GB/T 6435-2014 in China (weight reduction of feed after drying). Crude protein was detected by Primacs100 analyzer (Skalar, Dutch) as the standard of GB/T 18868-2002 in China (crude protein = total − *N* × 6.25). Crude lipid was detected by an XT15 extractor (ANKOM, USA) according to the standard of GB/T 6433-2006 in China (weight reduction of feed after extraction by petroleum ether). Ash was detected by burning at 550°C according to the standard of GB/T 6438-2007 in China (weight reduction of feed after fully burning) [17]. The determination of cholesterol content was followed by GB 5009.128-2016 in China. Briefly, 1.0 g samples were saponified by anhydrous ethanol-60% potassium hydroxide in solution (3 : 1, v : v) and extracted with petroleum ether and anhydrous ether (1 : 1, v : v). After the extracts were concentrated to dryness, it was dissolved in anhydrous ethanol, and detected by gas chromatography.

### 2.4. Hemolymph Biochemical Parameters

The hemolymph biochemical indicators include triglyceride (TG, item no. A110-1-1), total cholesterol (T-CHO, item no. A111-1-1), low-density lipoprotein cholesterol (LDL-C, item no. A113-1-1), high-density lipoprotein cholesterol (HDL-C, item no. A112-1-1), and malonaldehyde (MDA, item no. A003-1-2) were determined [18]. The activities of hemolymph superoxide dismutase (SOD, item no. A001-3-2), phenol oxidase (PO, item no. A136-1-1), and lysozyme (LZM, item no. A050-1-1) were analyzed [19]. All the hemolymph parameters were determined by commercial kits (Nanjing Jiancheng Bioengineering Institute, China) following the instructions and monitoring the absorbance changes by a full-wavelength microplate reader (Thermo, Multiskan GO 1510, USA) [20].

### 2.5. Hepatopancreas Biochemical Parameters

The activities of aspartate aminotransferase (AST, item no. C010-2-1), alanine transaminase (ALT, item no. C009-2-1), and alkaline phosphatase (AKP, item no. A059-2-2) were determined [21]. The hepatopancreas protease (item no. A080-2-2), amylase (item no. C016-1-1), and lipase (item no. A054-2-1) activities were analyzed. All the hepatopancreas parameters were determined by commercial kits (Nanjing Jiancheng Bioengineering Institute, China) following the instructions and monitoring the absorbance changes by a full-wavelength microplate reader (Thermo, Multiskan GO 1510, USA) [22].

### 2.6. Calculations and Statistical Analysis

The growth performance was calculated as follows [23]:

(1) $$\begin{array}{c} \text{Weight gain rate }\left(\text{WGR},\%\right)=\frac{100\times \left(\text{final body weight}-\text{initial body weight}\right)}{\text{initial body weight}},\\ \text{Specific growth rate }\left(\text{SGR},\%{\text{day}}^{-1}\right)=\frac{100\times \left(\text{Ln final body weight}-\text{Ln initial body weight}\right)}{\text{t}},\\ \text{Survival }\left(\%\right)=\frac{100\times \left(\text{final number of shrimp}\right)}{\left(\text{initial number of shrimp}\right)},\\ \text{Feed conversion ratio }\left(\text{FCR}\right)=\frac{\text{feed consumed }\left(\text{g}\right)}{\left(\text{final body weight}-\text{initial body weight}\right)},\\ \text{Condition factor }\left(\text{CF},\text{g }{\text{cm}}^{-3}\right)=\frac{100\times \text{final body weight}}{{\left(\text{body length}\right)}^{3}},\\ \text{Flesh content }\left(\%\right)=\frac{\text{muscle weight of shrimp }\left(\text{g}\right)}{\text{total weight of shrimp }\left(\text{g}\right)}, \end{array}$$

where *t* is the experimental duration in days.

The results were presented as the means with SEM. The data were compared between treatments by one-way analysis of variance (one-way ANOVA) using the SPSS 21.0 statistical software (SPSS, Chicago, IL, USA). When overall differences were significant (*P* < 0.05), Duncan's test was used to compare the mean values between individual treatments. Two-way ANOVA followed by Student-Newman-Keuls (SNK) multiple comparison test was conducted to investigate the interaction effects of sterol sources and levels. A probability value of *P* < 0.05 was deemed to be statistically significant.

## 3. Result

### 3.1. Effects of Dietary Sterol Sources on Growth Performance of Shrimp

As shown in Table 2, supplementation with higher levels of sterols helped improve the growth performance of shrimp. To be specific, the final body weight (FBW), weight gain rate (WGR), and specific growth rate (SGR) of shrimp significantly increased after supplementation of the feed with 2 g/kg cholesterol or mixed sterol sources (*P* < 0.05). There were no significant differences in growth performance indicators in shrimp fed with the HP diet compared to those fed with the HC and CP diets (*P* > 0.05). Also, the feed conversion ratio (FCR) significantly decreased after supplementation of the feed with 2 g/kg sterol sources in the HC, HP, and CP groups (*P* < 0.05). Survival was not correlated with either sterol source or level and did not differ significantly among the five groups (*P* > 0.05).

### 3.2. Effects of Dietary Sterol Sources on Body Composition and Physical Indicators of Shrimp

As shown in Table 3, the crude protein of shrimp decreased due to low dietary sterol levels. The crude protein content of shrimp fed with the HC and CP diets was significantly higher than the LC and LP groups (*P* < 0.05). The type of sterol sources had a significant effect on the crude lipid content, with a decline of which in LP and HP groups compared to the other three groups (*P* < 0.05 in the two-way ANOVA). The ash content of whole shrimp also increased when supplemented with higher dietary sterol levels, with the ash of shrimp fed the HC, HP, and CP diets significantly increased compared to those fed the LC and LP diets (*P* < 0.05). Neither the sterol source nor level had significant effects on the condition factor and flesh content of shrimp (*P* > 0.05).

### 3.3. Effects of Dietary Sterol Sources on Hemolymph Biochemical Parameters of Shrimp

The results of the hemolymph biochemical parameters of shrimp were shown in Table 4. Both sterol sources and levels as well as their interactions had significant effects on the hemolymph triglyceride (TG) and total cholesterol (T-CHO) of shrimp. Specifically, the hemolymph TG and T-CHO in shrimp fed the HC diets were significantly higher than in other groups (*P* < 0.05), while those in shrimp supplemented with dietary phytosterol showed a trend of reduction compared to the LC and HC groups. The sterol source had significant effects on hemolymph high-density lipoprotein cholesterol (HDL-C) content and malonaldehyde (MDA), with the former increased and the latter decreased in shrimp supplemented with dietary phytosterol (LP, HP, and CP groups) (*P* < 0.05). Neither the sterol source nor the level had significant effects on the hemolymph low-density lipoprotein cholesterol (LDL-C) content of shrimp (*P* > 0.05).

### 3.4. Effects of Dietary Sterol Sources on Nonspecific Immunity and Health Indicators in Hemolymph and Hepatopancreas of Shrimp

**Figure 1:**
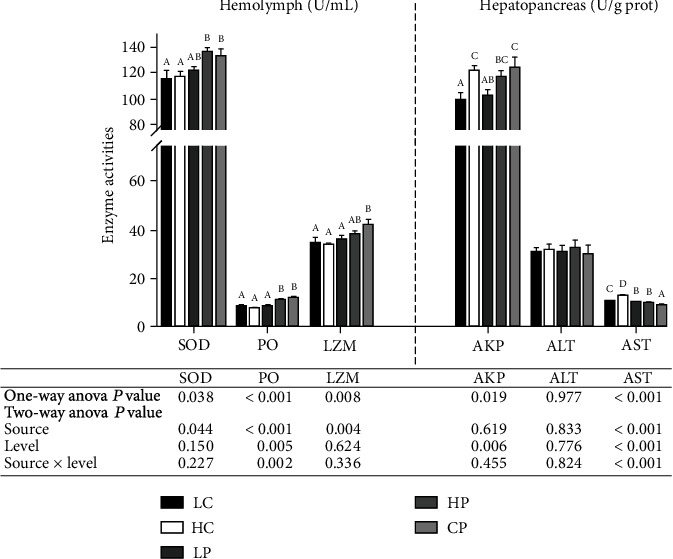
Effect of different sterol sources and levels on nonspecific immunity and health indicators in hemolymph and hepatopancreas of *L. vannamei*. Vertical bars represent the mean ± SEM (*n* = 3). Data marked with different letters differ significantly (*P* < 0.05) among groups based on Duncan's test. The lack of superscript letter indicates no significant differences among treatments. Two-way ANOVA followed by Student-Newman-Keuls (SNK) multiple comparison test was conducted to investigate the interaction effects of sterol sources and levels. SOD = superoxide dismutase (U/mL); PO = phenol oxidase (U/mL); LZM = lysozyme (U/mL); AKP = alkaline phosphatase (U/g); ALT = alanine transaminase (U/g); AST = aspartate aminotransferase (U/g).

The results of the enzyme activities related to nonspecific immunity and health are shown in Figure 1. The sterol source had significant effects on the hemolymph superoxide dismutase (SOD) and lysozyme (LZM) activities, with the SOD activity of shrimp fed the HP and CP diets as well as the LZM of shrimp fed the CP diet significantly increased compared to the cholesterol groups (*P* < 0.05). Both sterol sources and levels, as well as their interactions, had significant effects on the hemolymph phenol oxidase (PO) activity, which was boosted by dietary phytosterol and a higher level of sterol inclusion (*P* < 0.05). In hepatopancreas, the alkaline phosphatase (AKP) activity was shown to significantly increase in shrimp supplemented with 2 g/kg sterol level (*P* < 0.05). The interaction of sterol sources and levels had an impact on the aspartate aminotransferase (AST) activity, which significantly increased in shrimp fed the HC and LC diets compared to the other three groups (*P* < 0.05).

### 3.5. Effects of Dietary Sterol Sources on Digestive Enzyme Activities in Hepatopancreas of Shrimp

**Figure 2:**
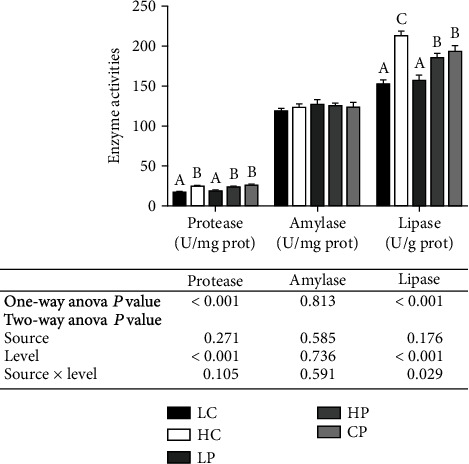
Effect of different sterol sources and levels on digestive enzyme activities in hepatopancreas of *L. vannamei*. Vertical bars represent the mean ± SEM (*n* = 3). Data marked with different letters differ significantly (*P* < 0.05) among groups based on Duncan's test. The lack of superscript letter indicates no significant differences among treatments. Two-way ANOVA followed by Student-Newman-Keuls (SNK) multiple comparison test was conducted to investigate the interaction effects of sterol sources and levels.

As shown in Figure 2, both protease and lipase activities of shrimp were mainly affected by sterol levels, and they could be improved by supplementing with 2 g/kg sterol sources (HC, HP, and CP diets) in the diet compared to lower sterol inclusion groups (1 g/kg sterol sources, LC and LP diets) (*P* < 0.05). It was also noted that the lipase activity was significantly higher in shrimp fed with the HC diet than that fed the HP and CP diets (*P* < 0.05). The amylase activity was not affected by the sterol source and level factors in this study (*P* > 0.05).

## 4. Discussion

For crustaceans, dietary cholesterol is essential for the synthesis of ecdysteroids and sesquiterpenoids, which regulate life activities including molting, growth, and reproduction [24]. However, shrimp are typically thought to be incapable of synthesizing sufficient cholesterol and require external intake, raising the challenges in shrimp farming: either higher feed costs or reduced growth [4]. Fortunately, some dietary lipid sources, including phytosterols and phospholipids, have been reported to function as cholesterol [5]. The phytosterols are primarily constituting of campesterol, stigmasterol, and *β*-sitosterol and have an extra hydrophobic carbon chain at the C-22 or C-24 position compared to cholesterol [25]. Similar to cholesterol, phytosterols form micelles with fat, bile acids, and cholesterol in the intestinal lumen and are absorbed by intestinal epithelial cells via sterol transporter Niemann-pick C1-like 1. The sterols are then esterified by acyl-CoA: cholesterol acyltransferase isoform 2 and combine with chylomicron to travel in the lymph and finally absorb by the liver via ApoE-dependent receptor [26]. Previous studies have reported that shrimp may be capable of converting phytosterols into cholesterol *in vivo* but the detailed biochemical process and bioavailability efficiency remains unknown [6, 7]. Also, phytosterols function in reducing cholesterol storage, alter lipid metabolism, and affect innate immunity as well as antioxidant capacity in some studies [12, 27–29]. Therefore, it is important to have a comprehensive understanding of the effects of dietary phytosterols on shrimp. In the present study, the growth performance significantly increased in shrimp supplemented with 2 g/kg cholesterol or mixed sterol sources, which indicated that increased intake of sterol level was beneficial to shrimp growth and that phytosterols can partially replace cholesterol. Moreover, the FCR in shrimp fed with the HC, HP, and CP diets was lower than those supplemented with 1 g/kg sterol level, implying that a higher sterol level (2 g/kg sterol level) could facilitate the nutrient utilized by shrimp and reduce feed consumption at the same growth performance. Besides, increasing the crude protein content of whole shrimp suggested that the higher sterol content positively affected the protein conversion of shrimp, which could be explained by the increased protease activity in HC and CP groups. In a previous study, the optimum cholesterol requirement of shrimp was around 1.7-2.7 g/kg based on the different indicators. However, when the dietary cholesterol was less than 0.8 g/kg, the FBW and WGR of shrimp showed a significant reduction compared to those fed higher and optimal cholesterol inclusion diets [7], which was similar to our results. Several studies have reported the safety and potential use of phytosterols in aquafeed, with no adverse effects on the growth of yellow croaker (*Larimichthys crocea*), Atlantic salmon (*Salmo salar* L), European sea bass (*Dicentrarchus labrax*), and gilthead sea bream (*Sparus aurata*) [12, 30–32] and growth-promoting effects in Bluntnose Black Bream (*Megalobrama amblycephala*), Tilapia (*Oreochromis niloticus*), and largemouth black bass (*Micropterus salmoides*) [33–36]. Although phytosterols can replace dietary cholesterol in shrimp feed, the molecular mechanisms by which phytosterols facilitate shrimp growth and the synergistic and competitive effects between phytosterols and cholesterol are still unclear, and more studies are needed to shed light.

Since phytosterols have the function of promoting sterol metabolism, they have predictable potential effects on lipid metabolism and nonspecific immunity in shrimp [26, 37]. In the present study, it is noted that whole-shrimp crude lipid content was mainly influenced by the sterol source, with a tendency for a decrease in lipid content caused by dietary phytosterols. Furthermore, the hemolymph TG and T-CHO content were affected by the interaction of sterol source and levels. To be specific, supplementing the feed with a high level of phytosterol significantly reduced the TG and T-CHO content. Studies have also shown that phytosterols are capable of reducing T-CHO, TG, and LDL-C concentrations by hepatic very low-density lipoprotein (VLDL) secretion or converting to bile acids [38, 39]. These lipid-lowering effects may be mediated by inhibiting translation levels of hepatic peroxisome proliferator-activated receptor alpha (PPAR-*α*) and hepatic fatty acid synthetase (FAS) [18, 40, 41]. Therefore, the supplementation of 2 g/kg phytosterols have been resulting in a cholesterol-lowering effect in *Litopenaeus vannamei* and how this effect affects the lipid metabolic processes in shrimp still needs further study.

In addition to growth and metabolism, the effect of dietary phytosterols on the nonspecific immunity of *Litopenaeus vannamei* deserves to be studied, as relevant studies are still missing. Different from vertebrates, shrimp fights against pathogens by innate immunity, in which prophenoloxidase, alkaline phosphatase (AKP), and lysozyme (LZM) play a remarkable role [42, 43]. The prophenoloxidase is capable of generating a vast amount of biologically active phenol oxidase (PO) through a cascade reaction and further mediates the synthesis of melanin [44, 45]. Then, melanin functions inhibit the growth and transmission of pathogens, and its intermediate metabolite quinone can cause toxic effects on pathogens, ultimately protecting the shrimp from invasion [46, 47]. It is also reported that LZM and AKP act as antibacterial enzymes in shrimp [48–50]. In the present study, the LZM activity was mainly affected by the sterol source rather than sterol levels, and high dietary phytosterol and mixed sterol sources showed a positive effect on the nonspecific immunity of shrimp. While the AKP activity was primarily influenced by sterol levels, the inclusion of 2 g/kg sterol levels boost the AKP activity in HC, HP, and CP groups compared to the LC groups. Besides, the PO activity was interactively influenced by sterol source and levels, with a significant increase in HP and CP groups, similar to the results of LZM. In a previous study, dietary phytosterols were reported to improve intestinal health, immunity, and anti-inflammatory activity of piglets [11]. Further, a diet rich in phytosterols is capable of reducing the risk of cardiovascular problems and cancer [51]. In some aquatic species, a diet supplemented with phytosterols had no effects on the immune response to gilthead sea bream and European sea bass [31, 32], suggesting that the tolerance and utilization of animals to phytosterols is species-specific. On the other hand, the antioxidant system also plays a vital role in supporting the immune response and preventing oxidative stress [52]. Usually, malondialdehyde (MDA) is used as a biomarker of oxidative damage in animals [53]. In the present experiment, the sterol sources have a main effect on hemolymph MDA content, with the MDA being lower in shrimp supplemented with phytosterols (HP, CP, and LP groups) compared to the LC and HC groups. In contrast to MDA content, the superoxide dismutase (SOD) activity in shrimp fed with HP and CP diets significantly increased compared to the LC and HC groups. These results suggest that dietary phytosterols help reduce oxidation damage and maintain shrimp health by activating SOD activity [54]. A previous study has shown that dietary phytosterols could reduce oxidative stress in the serum and liver of weaned piglets by stimulating SOD activity, and similar results were also reported in Sprague–Dawley rats [55, 56]. Not coincidentally, dietary phytosterols have been reported to increase serum and liver glutathione peroxidase activity and reduce breast muscle MDA content in Partridge Shank chickens [57]. In an earlier study, phytosterols and their major components were considered antioxidants [58]. This may be attributed to the induction of mitochondrial uncoupling by phytosterols leading to the production of reactive oxygen species and the stimulation of the glutathione redox cycle and antioxidant system, which ultimately protects cells from oxidative damage [59, 60]. In the future, the transcription and translation of immune genes and bacterial challenge experiments will be conducted to further investigate the effect of phytosterols on nonspecific immunity.

## 5. Conclusion

The supplementation of 2 g/kg phytosterols in the diet did not affect the growth of shrimp but resulted in a cholesterol-lowering effect in *Litopenaeus vannamei*. Mixed sterol sources could improve the growth and nonspecific immunity of shrimp. This study revealed the effects of different sterol sources and levels on the growth and nonspecific immunity of shrimp and provided a basis for further exploration of the mechanism of phytosterol.

## Figures and Tables

**Table 1:** Composition and nutrient levels of diets (dry basis g/kg).

Ingredients	Diets				
	LC	HC	LP	HP	CP
Soy protein concentrate	264	264	264	264	264
Fishmeal	200	200	200	200	200
Wheat flour	236	236	236	236	236
Meat and bone meal	50	50	50	50	50
Brewer's yeast	20	20	20	20	20
Fish oil	32	32	32	32	32
Soybean oil	10	10	10	10	10
Soybean lecithin	20	20	20	20	20
Ca(H_2_PO_4_)_2_	30	30	30	30	30
Cellulose	39	38	39	38	38
Vitamin C	1	1	1	1	1
Cholesterol	1	2			1
Phytosterol^1^			1	2	1
Sodium alginate	20	20	20	20	20
Choline chloride	10	10	10	10	10
Ethoxy quinoline	1	1	1	1	1
Vitamin premix^2^	26	26	26	26	26
Mineral premix^3^	40	40	40	40	40
Total	1000	1000	1000	1000	1000
Nutrient levels^4^					
Moisture	60.8	61.5	63.1	62.4	61.8
Crude protein	345.7	339.6	338.8	343.6	340.7
Crude lipid	82.3	80.7	81.9	80.0	80.7
Ash	51.6	50.8	51.0	51.8	50.6
Cholesterol	1.56	2.54	0.51	0.49	1.51

^1^The phytosterol was provided by Guangdong Wei Lai Biotechnology Co., Ltd. (Guangzhou, China) and constituted 28.1% of campesterol, 17.2% of stigmasterol, 43.9% of *β*-sitosterol, and others. ^2^Each 1 kg of vitamin premix contains vitamin A 4,200,000 IU, vitamin D3 2,200,000 IU, vitamin E 35 g, vitamin K3 10 g, vitamin B1 4.5 g, vitamin B2 16 g, vitamin B6 9 g, vitamin B12 0.02 g, calcium pantothenate 28 g, folic acid 2.5 g, biotin 0.08 g, nicotinic acid 38 g, and inositol 145 g. ^3^Each 1 kg of mineral premix contains MgSO_4_·H_2_O 13 g, KCl 85 g, Met-Cu 3 g, FeSO_4_·H_2_O 1 g, ZnSO_4_·H_2_O 12 g, Ca(IO_3_)_2_ 0.06 g, Met-Co 0.16 g, and NaSeO_3_ 0.003 6 g. ^4^Nutrient levels were measured values.

**Table 2:** Growth performance of shrimp fed different diets.

Sterol source	Sterol level (g/kg)	Group	FBW	WGR	SGR	Survival	FCR
Cholesterol	1	LC	7.06 ± 0.19^a^	1251.42 ± 21.03^a^	4.33 ± 0.02^a^	84.00 ± 7.11	1.31 ± 0.04^b^
	2	HC	7.94 ± 0.28^b^	1431.09 ± 61.78^b^	4.54 ± 0.06^b^	84.66 ± 2.49	1.15 ± 0.06^a^
Phytosterol	1	LP	7.19 ± 0.25^a^	1256.44 ± 54.14^a^	4.34 ± 0.06^a^	82.00 ± 2.82	1.28 ± 0.04^b^
	2	HP	7.56 ± 0.35^ab^	1359.07 ± 57.00^ab^	4.46 ± 0.06^ab^	82.66 ± 4.98	1.25 ± 0.04^ab^
Mixed sterol sources	2	CP	8.12 ± 0.36^b^	1462.36 ± 69.60^b^	4.57 ± 0.07^b^	85.33 ± 4.98	1.13 ± 0.05^a^
*P* value			0.020	0.009	0.009	0.951	0.019
Two-way ANOVA *P* value							
Source			0.336	0.302	0.322	0.780	0.255
Level			0.014	0.005	0.004	0.848	0.023
Source × level			0.243	0.348	0.359	1.000	0.104

Data represent mean ± SEM of three replicates (*n* = 3). Values in the same line with different letters are significantly different (*P* < 0.05) based on Duncan's test. The lack of superscript letter indicates no significant differences among treatments. Two-way ANOVA followed by Student-Newman-Keuls (SNK) multiple comparison test was conducted to investigate the interaction effects of sterol sources and levels. FBW = final body weight; WGR = weight gain rate; SGR = specific growth rate; FCR = feed conversion ratio.

**Table 3:** Body composition and physical indicators of shrimp fed different diets.

Sterol source	Sterol level (g/kg)	Group	Moisture	Crude protein	Crude lipid	Ash	Condition factor	Flesh content
Cholesterol	1	LC	73.83 ± 1.18	17.69 ± 0.99^a^	1.14 ± 0.11	2.90 ± 0.19^a^	0.59 ± 0.03	52.83 ± 1.64
	2	HC	72.46 ± 1.33	20.14 ± 0.85^bc^	1.28 ± 0.13	3.61 ± 0.14^b^	0.63 ± 0.02	54.21 ± 1.58
Phytosterol	1	LP	73.67 ± 0.84	18.06 ± 0.43^a^	0.98 ± 0.01	2.94 ± 0.07^a^	0.60 ± 0.04	52.63 ± 1.47
	2	HP	73.19 ± 0.83	18.73 ± 0.43^ab^	1.06 ± 0.03	3.37 ± 0.16^b^	0.62 ± 0.03	53.74 ± 1.27
Mixed sterol sources	2	CP	72.34 ± 0.72	20.61 ± 0.58^c^	1.17 ± 0.10	3.65 ± 0.06^b^	0.63 ± 0.02	55.14 ± 0.71
*P* value			0.498	0.007	0.071	<0.001	0.765	0.404
Two-way ANOVA *P* value								
Source			0.797	0.140	0.045	0.274	0.979	0.601
Level			0.226	0.010	0.127	<0.001	0.286	0.232
Source × level			0.546	0.104	0.636	0.164	0.812	0.894

Data represent mean ± SEM of three replicates (*n* = 3). Values in the same line with different letters are significantly different (*P* < 0.05) based on Duncan's test. The lack of superscript letter indicates no significant differences among treatments. Two-way ANOVA followed by Student-Newman-Keuls (SNK) multiple comparison test was conducted to investigate the interaction effects of sterol sources and levels.

**Table 4:** Hemolymph biochemical parameters of shrimp fed different diets.

Sterol source	Sterol level (g/kg)	Group	TG	T-CHO	LDL-C	HDL-C	MDA
Cholesterol	1	LC	2.26 ± 0.08^b^	1.67 ± 0.05^b^	1.29 ± 0.01	0.031 ± 0.001^a^	12.88 ± 0.83^c^
	2	HC	2.65 ± 0.06^c^	2.34 ± 0.03^c^	1.33 ± 0.02	0.030 ± 0.001^a^	11.21 ± 0.77^b^
Phytosterol	1	LP	2.10 ± 0.07^ab^	1.45 ± 0.02^a^	1.28 ± 0.04	0.035 ± 0.002^b^	8.68 ± 0.41^a^
	2	HP	2.08 ± 0.04^a^	1.36 ± 0.02^a^	1.25 ± 0.03	0.036 ± 0.002^b^	8.75 ± 0.46^a^
Mixed sterol sources	2	CP	2.19 ± 0.09^ab^	1.41 ± 0.07^a^	1.28 ± 0.02	0.040 ± 0.002^c^	8.14 ± 0.73^a^
*P* value			<0.001	<0.001	0.209	<0.001	<0.001
Two-way ANOVA *P* value							
Source			<0.001	<0.001	0.195	<0.001	<0.001
Level			0.006	<0.001	0.939	0.989	0.121
Source × level			0.003	<0.001	0.101	0.405	0.095

Data represent mean ± SEM of three replicates (*n* = 3). Values in the same line with different letters are significantly different (*P* < 0.05) based on Duncan's test. The lack of superscript letter indicates no significant differences among treatments. Two-way ANOVA followed by Student-Newman-Keuls (SNK) multiple comparison test was conducted to investigate the interaction effects of sterol sources and levels. TG = triglyceride; T-CHO = total cholesterol; LDL-C = low-density lipoprotein cholesterol; HDL-C = high-density lipoprotein cholesterol; MDA = malonaldehyde.

## Data Availability

The data that support the findings of this study are available on request from the corresponding author. The data are not publicly available due to privacy or ethics.
